# Asian Schistosomiasis: Current Status and Prospects for Control Leading to Elimination

**DOI:** 10.3390/tropicalmed4010040

**Published:** 2019-02-26

**Authors:** Catherine A. Gordon, Johanna Kurscheid, Gail M. Williams, Archie C. A. Clements, Yuesheng Li, Xiao-Nong Zhou, Jürg Utzinger, Donald P. McManus, Darren J. Gray

**Affiliations:** 1 QIMR Berghofer Medical Research Institute, Herston, QLD 4006, Australia; Catherine.Gordon@qimrberghofer.edu.au (C.A.G.); Yuesheng.Li@qimrberghofer.edu.au (Y.L.); Don.McManus@qimrberghofer.edu.au (D.P.M.); 2 Department of Global Health, Research School of Population Health, Australian National University, Acton, ACT 2601, Australia; Johanna.Kurscheid@anu.edu.au; 3 School of Public Health, University of Queensland, Herston, QLD 4006, Australia; g.williams@sph.uq.edu.au; 4 Faculty of Health Sciences, Curtin University, Bentley, WA 6102, Australia; archie.clements@curtin.edu.au; 5 Telethon Kids Institute, Nedlands, WA 6009, Australia; 6 Center for Disease Control and Prevention, National Institute for Parasitic Diseases, Shanghai 200025, China; zhouxn1@chinacdc.cn; 7 Swiss Tropical and Public Health Institute, CH-4002 Basel, Switzerland; juerg.utzinger@swisstph.ch; 8 University of Basel, CH-4003 Basel, Switzerland

**Keywords:** Asia, control, elimination, epidemiology, *Schistosoma japonicum*, *Schistosoma malayensis*, *Schistosoma mekongi*, schistosomiasis

## Abstract

Schistosomiasis is an infectious disease caused by helminth parasites of the genus *Schistosoma.* Worldwide, an estimated 250 million people are infected with these parasites with the majority of cases occurring in sub-Saharan Africa. Within Asia, three species of *Schistosoma* cause disease. *Schistosoma japonicum* is the most prevalent, followed by *S. mekongi* and *S. malayensis*. All three species are zoonotic, which causes concern for their control, as successful elimination not only requires management of the human definitive host, but also the animal reservoir hosts. With regard to Asian schistosomiasis, most of the published research has focused on *S. japonicum* with comparatively little attention paid to *S. mekongi* and even less focus on *S. malayensis*. In this review, we examine the three Asian schistosomes and their current status in their endemic countries: Cambodia, Lao People’s Democratic Republic, Myanmar, and Thailand (*S. mekongi*); Malaysia (*S. malayensis*); and Indonesia, People’s Republic of China, and the Philippines (*S. japonicum*). Prospects for control that could potentially lead to elimination are highlighted as these can inform researchers and disease control managers in other schistosomiasis-endemic areas, particularly in Africa and the Americas.

## 1. Introduction

Schistosomiasis is a parasitic disease caused by blood flukes of the genus *Schistosoma.* Six species of schistosomes infect humans: *Schistosoma mansoni* (occurring in Africa, South America, the Caribbean, and the Middle East), *S. haematobium* (mainly occurring in Africa and the Middle East, with recent autochthonous transmission observed in Corsica, France), *S. intercalatum* and *S. guineensis* (two rare species confined to a few countries in Central Africa), *S. japonicum* (Asia), and *S. mekongi* (Mekong Delta including Cambodia, Lao People’s Democratic Republic (Lao PDR), and previously Thailand whose current status is transmission interruption) [1,2,3]. A seventh species, *S. malayensis*, which is thought to be closely related to *S. mekongi*, is endemic in Malaysia [4]. In this review, we focus on only those species currently occurring in Asia: *S. japonicum*, *S. mekongi*, and *S. malayensis*, which cause intestinal schistosomiasis. The three Asian schistosomes are all zoonotic, whereas the remaining species infecting humans are generally considered human-only parasites, with some notable exceptions [5,6,7,8,9,10].

**Figure 1:**
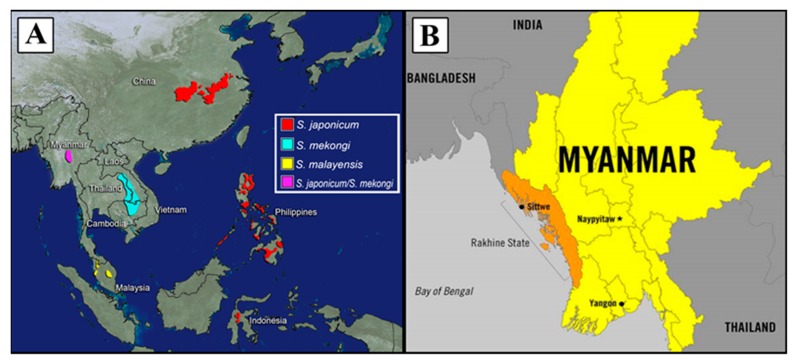
(**A**) Map of Southeast Asia showing the location of endemic areas for schistosomiasis, including a focus in central Myanmar. (**B**) Map of Myanmar highlighting the state of Rakhine.

Schistosomiasis has a long history in Asia with the first descriptions and reports of the disease in modern times appearing in the early 1900s, although it is thought to have been endemic for at least 400 years earlier in Japan, and at least 2200 years ago in the People’s Republic of China (P.R. China) after the discovery of *S. japonicum* eggs in a mummy [11,12,13,14]. To date, Japan is the only country in Asia to have eliminated schistosomiasis, while Thailand is awaiting verification of transmission interruption by the World Health Organization (WHO) [15]. Currently, schistosomiasis is endemic in six Asian countries: P.R. China, the Philippines, Indonesia, Lao PDR, Cambodia, and Malaysia, and is emerging in a seventh—Myanmar (Figure 1) [16]. Considerable progress in control has been made in recent decades, largely through praziquantel-based preventive chemotherapy (i.e., periodic administration of praziquantel to entire at-risk populations without prior diagnosis). However, preventive chemotherapy alone is insufficient to break the transmission cycle. Lack of safe water, poor sanitation, inadequate hygiene practices, limited health education, and the presence of animal reservoirs are known barriers to the elimination of schistosomiasis from a region [17]. Old challenges remain while new ones emerge, requiring a comprehensive, multi-sectoral, and multifaceted approach across the region to control this disease, and to reach the desired goal of elimination by 2030 [17].

The aim of this review is to provide an overview of the current status of schistosomiasis in Asia, with a particular focus on endemic countries in the region and the unique challenges they face. Our review also aimed to identify current knowledge gaps and future research needs as the affected countries move toward the ultimate goal of control and elimination of this persistent and debilitating disease.

## 2. Parasite Features

The genus *Schistosoma* is a group of parasitic blood flukes, or flatworms, of the class Trematoda. Unique amongst the trematode class, schistosomes have separate sexes as adults, whereas all other trematodes are hermaphrodites. The Asian schistosomes discussed in this review are considered zoonotic, unlike schistosome species occurring elsewhere, which are largely human-only excepting some hybrid forms in Africa [5,6,9,10,18], and cases of *S. mansoni* infecting non-human primates in Africa and the Caribbean, and rats in Guadeloupe and Brazil [7,8]. *S. japonicum* is the most cosmopolitan, with 46 mammalian definitive hosts identified thus far, whereas *S. mekongi* has been found in dogs, and *S. malayensis* in rodents, specifically *Rattus muelleri* [19,20,21,22] (Table 1). Pigs have been experimentally infected with *S. mekongi*, but, to date, no natural infections have been identified in these hosts [23]. Morphologically, the eggs and adults of the three species are very similar; the eggs are ovoid with a small ‘nubby’ lateral spine (Table 1) [24].

### 2.1. Lifecycle

**Figure 2:**
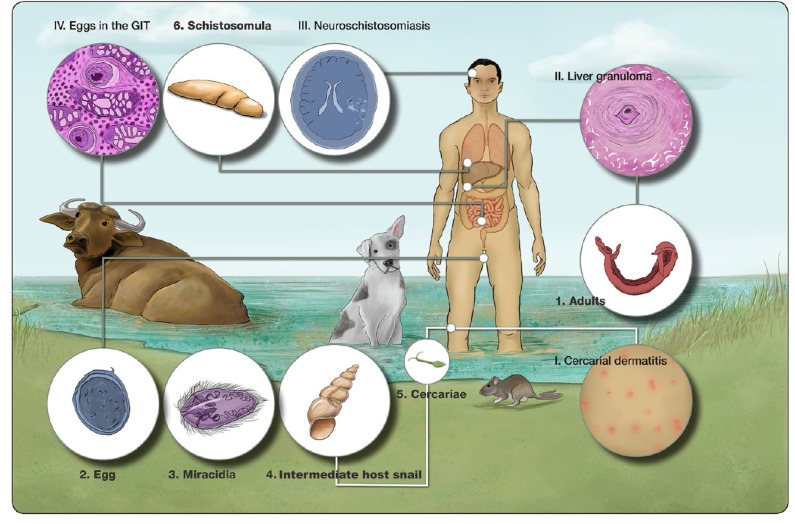
Schistosome lifecycle. Adult worms (**1**) reproduce sexually in the mesenteric veins surrounding the small intestine of the definitive mammalian host. Female worms deposit eggs (**2**), which are excreted in the feces. Upon contact with freshwater, the eggs hatch miracidia (**3**), which penetrate a snail intermediate host (**4**) and undergo asexual reproduction; this includes development of mother and daughter sporocysts, which produce cercariae (**5**). Cercariae exit the snail and swim around until they penetrate the skin of the mammalian definitive host, potentially causing cercarial dermatitis (**I**), shed their tail and become schistosomula (**6**). The schistosomula migrate through the body to the lungs before migrating and maturing to adult worms in the mesenteric veins. Chronic schistosomiasis occurs as the result of an immune reaction to the eggs resulting in granuloma formation in tissues where eggs are lodged. This most commonly occurs in the liver and spleen (**II**), which can result in hepatosplenomegaly and portal hypertension; in the walls of the intestine (**IV**) as eggs pass from the blood into the intestine; and less commonly in the brain (**III**), causing neuroschistosomiasis, characterized by a range of neurological symptoms. (Abbreviation: GIT, gastrointestinal tract).

The schistosome lifecycle is complex with an intermediate molluscan host, definitive host, and seven lifecycle stages involving both asexual and sexual reproductive phases (Figure 2). An in-depth understanding of social-ecological systems is required to grasp the spatial focality of schistosomiasis distributions [27].

#### 2.1.1. *S. japonicum*

*S. japonicum* is the most prevalent of the Asian schistosomes. It is endemic in P.R. China, the Philippines, and small foci occur in Indonesia. There are 46 known mammalian definitive hosts of *S. japonicum,* although water buffalo and cattle have previously been shown to be the major reservoirs of infection [19,28,29]. *S. japonicum* was first identified in Japan in 1901, whereas the last new human case was recorded there in 1977. *S. japonicum* parasites in P.R. China and the Philippines have distinct genetic differences, resulting from geographic isolation over time. In general, the strain of *S. japonicum* in P.R. China is more virulent than the parasite in the Philippines; additional genetically variant geographic isolates are known to be present in both countries [30,31,32].

#### 2.1.2. *S. mekongi*

*S. mekongi* was first identified in 1857 [33]. While morphologically very similar to *S. japonicum*, *S. mekongi* differs in a number of characteristics that indicate it is a distinct species. These differences include the morphology of the testis and ovary in adult worms [34] and the eggs of *S. mekongi* are smaller and more round than those of *S. japonicum* (Table 1) [35]. Morphological differences in the miracidial stage of the two species are also apparent [35]. Early genetic studies showing electrophoretic enzyme variation indicated sequence differences between *S. japonicum* and *S. mekongi* [36].

Apart from human infection, *S. mekongi* has only been identified naturally in dogs, although there have been successful laboratory infections of pigs. The intermediate host of *S. mekongi* is *Neotricula aperta* (previously *Lithoglyphopsis aperta*), and studies have found *Oncomelania* spp. snails to be refractory to infection with *S. mekongi* [34].

#### 2.1.3. *S. malayensis*

As with the other Asian schistosomes, *S. malayensis* is zoonotic and is primarily a parasite of the rodent *R. muelleri* (Table 1). *S. malayensis* is a sister species to *S. japonicum*, as is *S. mekongi*, to which it is more closely related [24]. Early studies identified the intermediate host of *S. malayensis* as *Robertsiella karporensis* [4,24].

### 2.2. Clinical Features

There are three clinical stages of schistosome infection. The initial early stage, a second ‘silent’ phase, also known as Katayama fever (or Katayama syndrome, named after the prefecture in Japan where it was first identified), and the third ‘chronic’ stage [1,37]. As the average life of an adult schistosome is 10 years and may be as long as 30 years, assuming no treatment, chronic infection can be lifelong [38,39].

The initial disease phase begins as a skin rash caused by an immune reaction to the penetrating cercariae (Figure 2, I). After penetrating the definitive host, the cercariae transform into schistosomula, which migrate to the lungs where they can cause pulmonary schistosomiasis, characterized as small nodules on a chest x-ray and a dry cough in the infected individual. After the lungs, the worms migrate to the venus plexus of the intestine, where they mature and pair up, reproducing sexually (Figure 2, 1). There is little immune response generated against the adult worms; thus, the second silent stage lasts for six to eight weeks post-infection when eggs begin to be produced. At this point, the acute Katayama fever begins, manifesting as fever, cough, rash, abdominal pain, nausea, diarrhea, and eosinophilia. Acute disease is more commonly seen in naïve persons, whereas chronic disease, the third phase, is more likely to occur in individuals resident in schistosome-endemic areas. Chronic disease occurs due to retention of eggs in the liver, spleen, and intestinal walls, and is the result of an immune response generated against the eggs, which causes granuloma formation in the various tissues (Figure 2, II and III). This can result in hepatosplenomegaly, portal hypertension, abdominal pain, and bloody diarrhea. A rarer manifestation of disease is neuroschistosomiasis (Figure 2, IV), which causes neurological symptoms, such as seizures and headaches, due to a granulomatous response against eggs in the brain, appearing as lesions on scans [40,41,42,43]. Infection in children is associated with growth stunting and intellectual disability and, in adults, with a reduced ability to work [44,45].

### 2.3. Diagnostics

A number of diagnostics are available, including coproparasitological examination (CopE), as well as molecular and immunological diagnostics. CopE methods rely on direct detection and visualization of parasite eggs in feces and include the Kato-Katz (KK) thick smear procedure, which is a mainstay of control programs due to the relative ease of performing the test at low cost, although it lacks sensitivity in low-intensity infections [46,47] and FLOTAC [48,49]. Other diagnostic approaches include formal-ethyl acetate sedimentation-digestion (FEA-SD) [50], the Danish Bilharziasis Laboratory (DBL) technique [51,52], and the miracidial hatching technique (MHT) [53,54], which also tests egg viability. Molecular diagnostics rely on detection of parasite DNA in clinical samples, often stool but also in urine, blood, and saliva, and include loop-mediated isothermal amplification (LAMP) [55,56,57,58], conventional polymerase chain reaction (cPCR) [59,60], real-time PCR (qPCR) [28,61,62,63,64], and digital droplet PCR (ddPCR) [65,66,67]. Immunological diagnostics rely on detection of circulating parasite antigens or antibodies generated against parasite antigens. Antibody detection might lack specificity and generally does not distinguish between past and current infections. A study by Cai et al., however, suggested that an immunological test combining two antigens (SjSAP4 + Sj23-LHD) may be useful for monitoring schistosomiasis control programs in the Philippines [68]. The main immunological methods include the enzyme-linked immunosorbent assay (ELISA) [69,70,71,72] and the rarely used circumoval precipitation test (COPT) [73,74]. Comprehensive reviews of the current diagnostic methods for schistosomiasis were provided by Weerakoon et al. and Utzinger et al. [58,75].

Sensitive and specific diagnostic procedures are required to monitor the success or failure of schistosomiasis control programs as well as to determine whether control efforts have resulted in elimination. However, the most sensitive diagnostics, involving molecular or immunological techniques, can be expensive and require specialized facilities and equipment and trained personnel to perform the procedures [57,76].

### 2.4. Treatment

Laboratory studies and clinical trials have shown that praziquantel, a pyrazinoisoquinoline derivative, is a safe and highly efficacious oral drug that is active against all schistosome species, although it is less active against juvenile schistosomes compared with adult worms and eggs [77,78,79,80,81]. The effective clinical praziquantel dosage regimen is 60 mg/kg orally in divided doses over one day (3 × 20 mg/kg doses 4-hourly, or 2 × 30 mg/kg either 4- or 6-hourly) for *S. japonicum* and *S. mekongi* [77,78]. Praziquantel is also the mainstay for preventive chemotherapy for morbidity control of schistosomiasis. In the Philippines, the efficacy of a single dose of 40 mg/kg vs. 60 mg/kg was compared; 40 mg/kg was effective and better tolerated and thus 40 mg/kg was adopted for preventive chemotherapy [82]. A single dose is beneficial for large-scale administration as it does not require follow up treatment as occurs with split doses. Despite the reliance on 40 mg/kg for preventive chemotherapy programs, two doses of 60 mg/kg separated by two weeks is recommended by the Philippine government in case finding, i.e., eggs identified in the stool [82].

Treatment with praziquantel does not prevent reinfection [83] and is therefore relatively ineffective at interrupting the transmission cycle. Praziquantel is principally aimed at reducing the prevalence and intensity of infection and to control morbidity over the longer term. Some concern has been expressed that praziquantel-resistant schistosomes may develop, most likely in Africa [84,85], and there is thus a pressing need to develop new anti-schistosomal drugs [86] and other non-pharmaceutical interventions.

## 3. Epidemiology

Due to the requirement of an intermediate host snail, schistosomiasis is a focal disease, occurring in areas where snail habitats and susceptible transmitting snails are present. This means that village-level prevalence can be very high, whereas country and province prevalence can be low. Demographic factors such as age, sex, and occupation are strongly associated with risk of infection [87,88]. Open defecation remains a common phenomenon in schistosomiasis-endemic countries and is strongly associated with transmission.

Snail habitats generally occur in still or slow moving water bodies such as streams, lakes, dammed waterways, and rice fields. The susceptible snails also have a preference for vegetation and snail control measures can include the removal of this vegetation.

### 3.1. Mammalian Definitive Hosts

Of the 46 known *S. japonicum* hosts, bovines, particularly water buffalo, are considered the most important for transmission due to the high levels of schistosome eggs they excrete into the environment [89,90] and their predisposition to natural infection. Epidemiological studies conducted in the Poyang and Dongting Lake regions of P.R. China revealed that water buffaloes account for up to 75–80% of *S. japonicum* infections, and hence are considered to be the most important reservoir hosts [29,89,90,91]. In mountainous and hilly endemic areas, water buffaloes are frequently used for ploughing rice fields and, while rodents have been considered to be important reservoirs, Van Dorssen et al. posited that this may not be the case owing to low levels of egg output and questions around egg viability [92,93]. Other potentially important animal reservoir hosts for *S. japonicum* in P.R. China include goats, pigs, and dogs due to their close contact with humans and water [93,94].

In Indonesia, 13 mammalian species, mostly wild animals (wild rodents, wild pigs, wild deer, wild celedus, and wild civet cats), but also cattle, water buffalo, horses, and dogs, have been identified as susceptible hosts for *S. japonicum* [95]. The prevalence of *S. japonicum* in water buffalo (known also as carabao) in the Philippines has been reported to be as high as 80%, particularly in agricultural areas, and water buffalo are thought to be a major reservoir [96]. In both the Philippines and P.R. China, there are fewer cattle than water buffalo/carabao. Cattle are more susceptible to infection than water buffalo, likely due to their more recent introduction into Asia compared with water buffalo, which have co-evolved with *S. japonicum* for much longer. Although studies suggested that water buffalo exhibit some age-acquired resistance to infection and self-cure [28,97,98], there is still uncertainty regarding this phenomenon [99].

Due to the close genetic relationship between *S. japonicum* and *S. mekongi*, bovines could act as reservoir hosts of *S. mekongi* but, to date, this has not been demonstrated. A range of potential animal hosts have been examined for *S. mekongi*, but currently dogs are the only animal species that have been confirmed as natural hosts of this species [22].

In regards to control, little has been done to target definitive hosts of *S. japonicum*, with the exception of P.R. China, which has practiced both chemotherapy of bovines and removal of the animals, facilitated by mechanization of agriculture (i.e., replacing water buffalo with tractors) [16,29,89,100,101]. Without targeting intermediate host snails, re-infection of humans after treatment can occur almost instantaneously. Animals can also contribute to rebounding infections in areas where humans have been declared free of schistosomiasis [102]. In Lao PDR, chemotherapy of dogs against *S. mekongi* has been highlighted as a priority. This has been proposed as part of community-led initiatives to eliminate schistosomiasis that combine deworming with water, sanitation, and hygiene interventions: community-led school, water, sanitation, and hygiene (CL-SWASH) activities [103].

Animal vaccines against schistosomiasis have been developed and used in controlled trials [104,105,106,107]. Whereas none of the currently developed vaccines provide 100% immune protection (0–80% worm reduction in mice and baboons [108]; 41–51% in water buffalo [109]), they do induce a significant reduction in adult worms, decreased egg output, and stunting of adults. A knockdown in adult worm fecundity alone can have a huge impact on transmission. Modeling has shown that an animal vaccine with 75% efficacy will be required to ensure long-term control of schistosomiasis [110].

### 3.2. Molluscan Intermediate Hosts

The intermediate snail hosts of *S. japonicum* are amphibious and belong to the genus *Oncomelania,* with species dependent on geographical location. Studies investigating the susceptibility of snails from different geographic locations to cercariae from disparate locations have produced mixed results, indicating a certain amount of genetic drift of the parasite [111,112]. Although Japan has successfully eliminated schistosomiasis, the requisite intermediate host snail species, *Oncomelania hupensis nosphora* and *O. hupensis formosana*, are still present [113].

In P.R. China, four sub-species of the intermediate host *Oncomelania hupensis* have been identified based on morphological and molecular characteristics [114,115,116], each with different growth rates, population genetics, and ecological niches. Control measures have been successful in reducing the populations of *O. h. guangxiensis* and *O. h. tangi* [13] leaving *O. h. hupensis* and *O. h. robertsoni* as the dominant sub-species [114]. Of these, *O. h. hupensis* is the most widely distributed [114]. Few studies have reported *S. japonicum* infection rates in *Oncomelania* snails in endemic regions of P.R. China in the last decade. One study in the marshland regions along the Yangtze River [114] examined more than 70,000 snails over a 15-year period (2001–2015) and found an overall prevalence of 0.05% with no new infections since 2007 [117]. An earlier study reported a decline from 0.88% in 2009 to zero in 2012 in Jiangling county, Hubei province [118]. According to a 2017 WHO meeting report on Asian schistosomiasis, an active sentinel surveillance program, conducted in 2016 in areas where transmission was considered interrupted or under control, also failed to identify infected snails [119]. However, the same report indicated that in two other studies undertaken in 2012 and 2017, in four and seven provinces, respectively, infected snails were found based on LAMP analysis, but no infection was recorded by microscopy, although actual infection rates for the LAMP analysis were not provided [119]. As snail infection rates continue to decline, consistent and highly sensitive diagnostic tests, such as LAMP, will be required to provide accurate information [75,120].

In the Philippines, the sole intermediate host snail for *S. japonicum* is *O. h. quadrasi*. It is amphibious but prefers an aquatic environment, such as wet soil surfaces, swamps, rice fields, ponds, and stream banks, thus making chemical snail control difficult due to the risk of contaminating the water or food source [17]. The current status of *S. japonicum* infection in the snail intermediate hosts in the Philippines is poorly understood. One study, conducted in Samar province, found a mean infection rate of 1.09% across 147 sites with higher infection among snails located in irrigated compared to rain-fed villages [121]. More recent data from 2013 to 2015 indicate infection rates of less than 2% in most endemic provinces, although Northern Samar was found to have a prevalence of above 12% [76].

In Indonesia, the intermediate snail host of *S. japonicum* is *O. h. lindoensis*, which is located focally around Lake Lindu. In 2011, the prevalence of infected *O. h. lindoensis* snails in Lindu Valley was 3.6% and 4.0% in Napu Valley, although the prevalence has fluctuated between 0 and 13.4% in Napu Valley and between 0% and 9.1% in Lindu Valley since 2005 [95]. Prevalence of infected snails in Bada Valley appears to be much lower, with a survey conducted in 2010 identifying a prevalence of only 1% (3 among 299 snails sampled) [122].

The intermediate hosts of *S. mekongi* are *Neotricula* spp., and endemic areas are closely associated with the Mekong Delta where these snails occur. The prevalence of infected snails in Lao PDR was quite low, 0.01% on the Mekong Islands [123] and 0.22% in Khong District, although the snail density was quite high [124]. *Neotricula aperta* snail density in Thailand decreased between 2005 and 2011 in the downstream area of the Nam Theun 2 hydroelectric dam, which began operation in 2010 [125]. A similar decrease in *Oncomelania* snails in low-land areas was initially seen in P.R. China after the building of the Three Gorges Dam [126]. However, snail density began to increase in 2011 after initially decreasing in the years immediately after completion of the dam in 2003. A similar trend may eventually be seen in Thailand with the Nam Theun 2 Dam.

Control of intermediate host snails is an important aspect of schistosomiasis control programs, particularly as the stage of the lifecycle occurring in snails is asexual and involves an exponential increase in parasite numbers. Snail control measures previously implemented in Asia have involved environmental modification and chemical mollusciciding. The snails live in vegetation around rivers and lakes and removal of this vegetation can lead to removal of the snails themselves. This method was used with great success in Japan in combination with mollusciciding. In Japan, the most common method of environmental modification was the use of concreting canals where the snails lived [113]. This method is more difficult to implement in areas where the snail habitats are rice fields or marshland. Early reports from Mindanao in the Philippines showed that the method of farming (weeding and ploughing) practiced in Mindanao reduced snail habitat on the rice fields, and thus snails were primarily found in swampland, at least when intensive farming was practiced [127]. Changing land use in Japan from rice crops to either housing or fruit trees, which did not require flood irrigation and thus no longer provided snail habitats, was an important feature for control.

Mollusciciding has also been used in P.R. China, Indonesia, and the Philippines, although to a limited degree in the latter two countries. Environmental contamination with chemical molluscicides is an important issue, and a number of previously used compounds have been abandoned due to these concerns and as a result of the damage they cause to the environment. In the schistosomiasis foci in Indonesia, the snail habitats occur close to the Lore Lindu National Park, which precludes the use of molluscicides [128] and environmental modification. Early molluscicides included lime, which proved inefficient, calcium cyanamide, and, later, sodium pentachlorohenate (Na-PCP), which was eventually stopped in all countries due to environmental toxicity [129]. In P.R. China, the molluscicide of choice has been niclosamide, used in two different formulations: (1) a 50% niclosamide ethanolamine salt wettable powder and (2) a 4% niclosamide ethanolamine powder [106,130]. Both formulations resulted in substantial, but not 100%, killing of snails, which meant that mollusciciding needed to be performed more than once a year.

*Neotricula* spp., the intermediate host snails of *S. mekongi,* exhibit a different ecological niche than those transmitting *S. japonicum* in that, rather than being present in marshlands and rice fields, these snails are primarily found in shallow areas of rivers (particularly the Mekong River) and tributaries. Thus, snail control for *S. mekongi* has largely been deemed infeasible [17], although ecological management of snail habitats upstream of human habitation, as occurs in P.R. China, should be explored. Instead, preventive chemotherapy, along with improved WASH is practiced. Lao PDR has previously used niclosamide for snail control, although this did not significantly impact the numbers of snails in the treatment areas [124].

### 3.3. Environment

The majority of environmental factors associated with *S. japonicum* transmission are related to distance to a snail habitat or those that influence snail habitats, such as the building of dams. The majority of *S. japonicum*-endemic zones are within 1 km of water bodies such as rivers, lakes, or wetlands [131,132]. Environmental factors that influence snail habitat include land cover, particularly the presence of flooded agricultural land [133,134], seasonal land surface temperature (LST), elevation, and rainfall [131].

In P.R. China, endemic areas occupy three different geographical landscapes: (1) marshland and lake areas, (2) mountainous and hilly areas, and (3) water network areas. Of these, marshland and lake areas are characteristic of the major endemic foci for *S. japonicum*, and might account for 95% of the snail habitats [17,87]. *Oncomelania* snails survive best at areas of low elevation—one of the potential environmental factors associated with high prevalence of snails in marshy areas. A study on snail habitats in mountainous and hilly areas identified a maximum elevation of 2300 m above sea level for snail survival [131]. The same study identified an ideal LST of ≥22.7 °C and a normalized difference vegetation index (NDVI) of ≥0.446 in the mountainous areas. Distance from the nearest stream was also important, as the *Oncomelania* snails are amphibious; yet, require water to survive. A distance of ≤1000 m from the nearest stream was found to be ideal for snail habitats [131].

As the marshy and lake areas are categorized by the presence of water bodies, more areas are available for the snails to exist. The area of these landscapes, which cover the four provinces of Hunan, Jiangxi, Anhui, and Hubei, is vast, complicating snail control in these locations [135]. Mountainous and hilly areas, located primarily in the western part of P.R. China in the provinces of Yunnan and Sichuan [131], account for approximately 5% of the remaining snail habitats [17]. The complex environmental conditions present in these areas make it difficult to control snail populations [131]. The third type of landscape are water network areas, mainly located around the Yangtze River, which account for <1% of snail habitats in endemic areas of P.R. China [17].

Local epidemic outbreaks and the geographic distribution of snail hosts are heavily influenced by flooding events caused by the Yangtze River as they facilitate snail dispersion to new localities such as rivers, lakes, and wetlands [136,137]. Large-scale water development projects [138], particularly the aforementioned Three Gorges Dam and the South-North Water Diversion project (SNWD), also influence the transmission and geographic distribution of schistosomiasis [139,140]. The SNWD plans to divert water from the Yangtze River to the North [141]. Climate prediction models have indicated that this project may result in the expansion of viable snail habitats for the main snail intermediate host *O. h. hupensis* as well as *O. h. robertsioni* and *O. h. guangxiensis* [141]. The Three Gorges Dam, begun in 2003 and completed in 2012, was built to decrease flooding events as well as to generate power. As a consequence, it has changed the ecology of the surrounding area and impacted the habitat of *Oncomelania* spp. snails. The decrease in flooding events has decreased the density of snails in some areas, although in others, the density appears unchanged, or is on the increase [126].

In the Philippines, more than 3000 bodies of water are thought to be infested with snails susceptible to *S. japonicum* infection: 80% in Mindanao, 18% in Visayas, and 2% in Luzon [76]. Endemic regions have no distinct dry season and are predominantly comprised of rice fields, where contact between humans and snails is maximized [11,82]. Environmental factors, such as close proximity to large perennial water bodies (PWB), LST, NDVI, and precipitation, influence *S. japonicum* infection prevalence differently in the three main regions of the Philippines [87]. As is the case in P.R. China, the distance from water is an important factor for snail habitats, with the prevalence of schistosomiasis in humans decreasing with distance to PWB [87]. Some differences exist between the three regions, with increased distance to PWB associated with decreased prevalence in Luzon and the Visayas, but not Mindanao, whereas LST increase only significantly associated with decreased prevalence in Luzon. Similarly increased precipitation was associated with higher prevalence in the Visayas but decreased prevalence in Mindanao [87]. A confounding factor may be the differences in average socioeconomic status between the three areas: people in Luzon tend to have higher socioeconomic status compared to schistosomiasis-endemic areas of the Visayas and Mindanao. Natural habitats of *O. h. quadrasi* include flood plains, forests, and swamps, whereas man-made habitats resulting from agricultural development are thought to be important habitats (e.g., drainage channels, roadside ditches, small canals, and drainage canals of irrigation works). These snails are generally found on banks but also occur in shallow water (depth <20 cm) [121]. *O. h. quadrasi* snails prefer areas shaded by vegetation where the temperature is relatively stable and cool.

Endemic regions of Indonesia are located in marshland areas around Lake Lindu and Napu and Bada valleys. Prevalence of *S. japonicum* in snails from this area ranged from 0 to 13.4% in the Lindu Valley and 0 to 9.1% in the Napu Valley, although human prevalence remained <1% as of 2006 [128].

*S. mekongi* transmission occurs in the Mekong Delta. In Khong and Mounlapamok districts in Lao PDR, 202 villages are situated along the Mekong River with 114 currently or previously endemic for schistosomiasis. The only villages with zero prevalence for *S. mekongi* are in parts of the river where the riverbed is sandy, which is not conducive to the intermediate host snail, or those villages that are more than 6 km away from the river [142].

Limited information is available regarding risk factors and snail intermediate hosts in Myanmar [16]. The current areas where schistosomiasis occurs are around Lake Inlay in Shan State, although a recent outbreak has occurred in Rakhine State on the Coast of the Bay of Bengal [143]. The wet season runs from May to October.

### 3.4. Transmission and Control

In P.R. China, transmission usually occurs across two distinct seasons [17], coinciding with the natural annual flooding events in the Yangtze River: firstly in April to June/July when flooding is at its peak, and secondly after the waters subside in September/October with transmission continuing until November [140]. Although environmental factors heavily influence the snail intermediate hosts, demographic factors and the presence of host reservoirs play a more significant role in human transmission of *S. japonicum*. Infection with *S. japonicum* is strongly associated with age, sex, and occupational exposure. Males aged 40 years and above who engage in fishing, farming, and herding are at greatest risk of infection [144]. Defecation into lake waters or marshlands by fishermen and grazing water buffalo facilitate the continuance of transmission.

**Figure 3:**
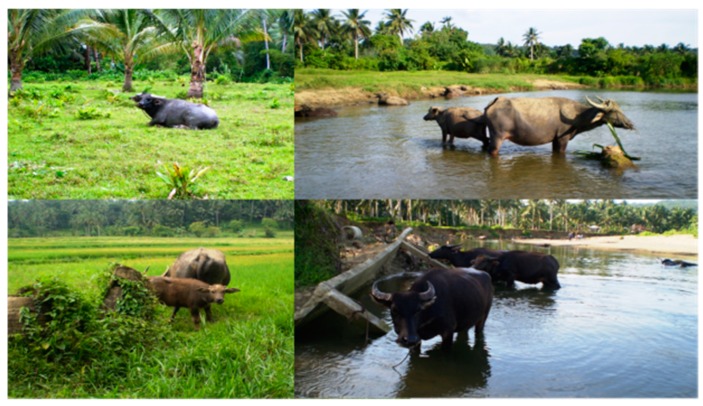
Water buffalo (carabao) in the Philippines are tethered in rivers, rice fields, and wallows—the same areas where the intermediate snail host for *S. japonicum* is also found. (Images from the Philippines, captured by C.A.G.).

**Figure 4:**
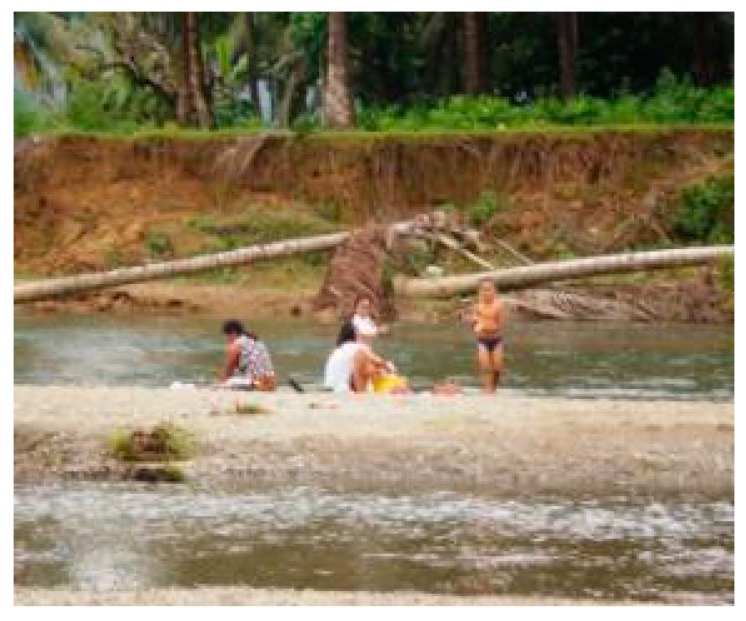
Washing and recreational uses of waterways are risk factors for contracting schistosomiasis. (Image from the Philippines, captured by C.A.G.).

In the Philippines, there is no distinct dry season on the main endemic islands of Leyte and Samar and the province of Mindanao. Hence, transmission is not as variable by season as in P.R. China and occurs all year round [82]. The most common crop in these endemic areas is rice, which provides contact between snails that live in the rice paddies, swamps, and streams, whereas water buffalo and cattle are used to work on the fields (Figure 3). In addition to farming, washing, and recreational use of rivers are associated with higher risk of infection (Figure 4).

Whereas 13 species of mammalian hosts have been identified in Indonesia, limited research has been undertaken on their involvement in transmission. Rodents of the genus *Rattus* have been suggested as the primary source of transmission, with a peak prevalence of 20% found in one endemic village [128]. Primary species thought to be involved in transmission are *R. exulans*, *R. hoffmani*, *R. chysocomus rallus*, *R. marmosurus*, and *R. celebensis* [128]. Reservations around the role that rodents can play in transmission were addressed earlier [92,93]. As in P.R. China, Indonesia experiences wet and dry seasons; hence, it is likely that schistosome transmission is also seasonal there, with increased transmission occurring in the wet season (November–March).

The transmission season for *S. mekongi* is matched with the lifecycle of the snail. During times of high water levels (2–3 m), the majority of available snails are young, while in times of low water (April–May; 10–60 mm), the snails have matured to adults capable of carrying the infection. Peak transmission of *S. mekongi* in Cambodia occurs between February and April coinciding with peak water use for fishing [17]; whereas in Lao PDR, the main transmission season occurs in April and May.

In Malaysia, the wet season differs between the southwest where the monsoon season is May–October, and the northeast where the monsoon season runs from November to March, and the typhoon season occurs from April to November. It is therefore difficult in the absence of yearly surveys to pinpoint when transmission in Malaysia might peak, but it is certainly influenced by rainfall brought by the monsoons and typhoons.

Preventive chemotherapy with praziquantel has been the mainstay of schistosomiasis morbidity control and, in addition to targeting mammalian and snail hosts mentioned earlier, efforts to control transmission have also included programs aimed at improving WASH and health education. Until relatively recently, the role of WASH in schistosomiasis control was limited [145,146,147,148], despite the strong association of the disease with poverty and poor sanitation. In 2012, the World Health Assembly (WHA) encouraged the incorporation of WASH into control and elimination strategies [149]. Due to the transmission dynamics of schistosomiasis, WASH primarily limits environmental contamination with schistosome eggs and reduces human contact with potentially infested waters [150]. Improvements in sanitation and access to clean water have been shown to reduce the risk of schistosome infection [151], and have the added benefit of reducing infection with other parasites such as soil-transmitted helminths [146]. The impact of WASH is, however, dependent on the setting [151]. For example, access to clean water is not considered to play a significant role in endemic areas where schistosome infections are attributed to occupational or recreational contact with water as opposed to water used for drinking or for everyday activities (e.g., laundry and bathing) [151]. Traditional WASH practices, such as handwashing, have little impact on schistosome infection as the parasite eggs excreted in stools are not infective to human or animal hosts. However, water use practices involving rivers, such as bathing and washing clothes, will increase risk of contact with the infectious cercariae. Hence, much of the WASH emphasis to date has focused on sanitation and sanitary behavior. A number of programs focusing on improving access to clean water and improved sanitation outside of schistosomiasis control are ongoing throughout schistosomiasis-endemic countries in Asia [152,153,154,155,156,157].

Health education is important not only for educating the public on risk reduction measures and changing behavior, but has also been found to facilitate diagnosis, surveillance, and treatment [157]. Through health education activities and water contact studies, many high-risk behaviors and at-risk populations have been identified [158]. This has enabled health messages to be tailored to specific groups, such as school-age children swimming in freshwater and farmers and fishermen, and has largely been aimed at methods of avoiding water contact and self-protection [158]. However, it can be difficult to change behavior in some groups, such as fishermen or farmers, due to the nature of their occupations [158]. The support of local and national governments, through implementing infrastructure such as public toilets and using sanitary containers for stool on fishing boats, is therefore important in these situations [117,159]. The vehicles used for health education messages are many-fold and include audio-visual (radio, television, film, drama, traditional opera, and exhibits), print media (poems, slogans, posters, magazines, and newspaper), and other daily articles such as printed shirts, towels, fans, and umbrellas, among others [158]. Health information can also be passively disseminated through the community by students, teachers, village leaders, and parents [158]. Although health education is considered an important component of an integrated control program for schistosomiasis [160,161,162,163], it needs to be thoroughly planned, targeted, trialed, and evaluated prior to implementation [158], and needs to be sustained over a long period of time in order to maximize effectiveness [164]. To date, health education has been a major focus in P.R. China. Elsewhere, health education has generally been combined with preventive chemotherapy and has played a limited role in schistosomiasis control and elimination programs [82,165].

Research is ongoing for the development of schistosomiasis vaccines targeting humans and, unlike the African schistosomes, the zoonotic nature of the Asian schistosomes allow for the targeting of animal hosts. With bovines, particularly water buffalo/carabao confirmed as the major reservoirs of *S. japonicum*, there is the rationale for the development and deployment of a transmission-blocking anti-*S. japonicum* vaccine targeting bovines [29,91,166,167]. The SjCTPI-Hsp70 vaccine is one of the most efficacious to date with an experimental efficacy of ~52% [101] and cluster-randomized controlled trials are currently being finalized to determine efficacy in natural settings. Vaccines may be the key for long-term sustainable control and elimination of schistosomiasis but research needs to be ongoing [167].

## 4. Current and Historical Status of Schistosomiasis in Asia

Notably, most prevalence estimates reported are based on microscopic detection of schistosome eggs in stool samples, usually using the KK thick smear technique [87,168,169]. However, numerous studies have demonstrated significantly higher prevalence when molecular detection methods have been applied on the same set of samples, with differences of up to 70% reported [28,60,63,170]. Prevalence is also largely influenced by the size of the sampled population. This is particularly relevant to the Philippines and Indonesia where funds are often limited, thus restricting the number of personnel available to interview and process collected samples. Another confounding factor, which is also common in P.R. China, is a fall in participation rates in many endemic areas, often referred to as treatment fatigue. Some communities in highly endemic areas have been participating in surveys for decades; hence, it is not surprising that these villagers have tired of the routine. In areas where prevalence has dropped significantly, the disease is no longer seen as a priority. Based on these factors, it is conceivable that the prevalence of schistosomiasis is considerably underestimated.

### 4.1. S. japonicum

The first reports of *S. japonicum* in P.R. China and the Philippines occurred around the same time in the early 1900s [171,172] and the epidemiology of the disease is similar in the two countries. The presence of the disease in Indonesia was first reported about three decades later, with an autochthonous infection in a 35-year-old male suffering from chronic schistosomiasis resulting in his death in 1937 [128].

#### 4.1.1. Japan

As indicated in the species name, *S. japonicum* was once found in Japan, the last reported human case was in 1977, and elimination of schistosomiasis was declared in 1996 [17]. There were a number of endemic areas in Japan pre-elimination, including Kofu basin, Fukuoka, and Saga prefectures, which appeared to have had the highest prevalence [173]. Due to a successful control program, transmission of the parasite no longer occurs there, although the requisite intermediate host snail species, *Oncomelania nosphora*, is still present [113]. In addition to the control initiatives implemented, modernization and socioeconomic development had a large impact on elimination of this parasite in Japan. Elimination of schistosomiasis in Japan occurred pre-praziquantel and the available drug at the time, stibnal, caused severe adverse events, which resulted in low treatment compliance. Thus, most control efforts focused on targetting the snail intermediate hosts and preventing transmission to humans.

Environmental modification in the form of concreting canals, where the *Oncomelania* snails bred, began in 1938 and was the primary method used [17]. In addition, the Japanese government purchased and buried snails from people in the endemic areas. Mollusciciding using lime, which proved to be inefficient; calcium cyanamide, which proved to be more efficient; and later Na-PCP were trialed. The use of Na-PCP was eventually stopped after 13 years due to its environmental toxicity. Hot water and flamethrowers were also used to kill snails, and proved effective in small areas. In addition, geese and firefly larvae were released into endemic fields to eat snails, although these measures proved unsuccessful [129]. Land use was changed, with increased urbanization meaning that many paddy fields were converted to housing, and in other areas, crops changed from rice to fruit trees; this precluded the use of flood watering and thus no longer provided areas for snail breeding [17]. Education of farmers to prevent the use of “night soil” as fertilizer was also performed, thus limiting environmental contamination with schistosome eggs from human feces. Bovines were replaced with horses, which can act as reservoir hosts but are less efficient transmitters, and other potential reservoirs such as wild populations of mice and dogs were controlled.

#### 4.1.2. P.R. China

In P.R. China, *S. japonicum* is predominantly found in areas along the middle and upper reaches of the Yangtze River Valley in the southern part of the country where the climate and environment are highly suitable for the propagation of *Oncomelania* snails. Endemic regions are concentrated in the lake regions and in the mountainous region in the western part of the country [131]. Until relatively recently, *S. japonicum* was endemic in 12 provinces, but due to political will and sustained efforts, primarily through snail control and preventive chemotherapy, five provinces have achieved transmission interruption. Of the remaining seven provinces, four (Sichuan, Yunnan, Jiangsu, and Hubei) achieved transmission control (prevalence <5% in humans and animals) by 2014, whereas Anhui, Jiangxi, and Hunan are still in the infection control stage (prevalence <1% in humans and animals) [174,175]. In 2012, an estimated 800,000 people were infected and 65 million people considered at risk [24]. In 2016, the number of reported cases had dropped considerably and was just over 77,000 [17]. Current human prevalence in most endemic villages is between 1% and 3% but among the high-risk population, such as those who have extensive contact with water, infection levels may still exceed 10% [17].

Control approaches in P.R. China have been extensive due to the strong commitment by the national government. Funds from a 10-year World Bank Loan Project (WBLP) implemented in the 1990s [176] were put toward control and prevention strategies for schistosomiasis, including preventive chemotherapy, snail control, and WASH interventions. The mainstay of schistosomiasis control is preventive chemotherapy with praziquantel. In P.R. China, preventive chemotherapy is primarily targeted at fishermen and boat people living within half a kilometer of schistosomiasis-infested water bodies and is administered biannually. Treatment in other high-risk populations is selective, based on the extent of water contact [16]. P.R. China is the only endemic country that also practices mass drug administration for bovines, which are treated annually. The government is replacing animals used for farming with tractors and is removing bovines [16,89,100]. Mollusciciding occurs annually and usually coincides with the onset of the transmission seasons. Ecological methods of snail control have been used in P.R. China, such as changing farming practices, submerging snail habitats, and placing black plastic film over banks post-mollusciciding [17]. P.R. China’s efforts to improve WASH began during the third phase of their national schistosomiasis control program (initiated in 2004), which focused on an integrated approach to control transmission [177]. WASH interventions mainly center on fishermen and boatmen [178] in the Poyang and Donting Lake regions and include supplying tap water and stool containers and building latrines close to boat anchoring points [159,179]. Health education has been an integral component of P.R. China’s schistosomiasis control program since its inception in the 1950s [164].

#### 4.1.3. Philippines

In the Philippines, *S. japonicum* is distributed throughout all three major island groups (Luzon, Mindanao, and Visayas), although the majority of cases occur in Mindanao and the Visayas [87,180] (Figure 1). Schistosomiasis is currently endemic in 28 of the country’s 80 provinces, mostly in Mindanao, with more than 12 million people estimated to be at risk and 2.5 million directly exposed [76]. Distribution of *S. japonicum* is more widespread in the Visayas compared to Luzon and Mindanao, where it is more focal [87]. Among endemic provinces, 10 are considered highly endemic (prevalence >5%), six moderately (1–4.9%), and 12 low or close to elimination levels (<1%) [76]. Prevalence studies conducted since 2000 indicate infection levels vary based on location. In 2000, Acosta et al. reported a prevalence of 60% in adolescent and young adults (aged 15–30 years) in three villages in Leyte [181]. In 2005, a cross-sectional survey in Western Samar Province reported a prevalence range of 0.7% to 47% [182], which is similar to that reported by Ross et al. in 2012 from Northern Samar [96]. A nationwide prevalence study in 2015 identified infection levels >5% in one province, 1–5% in 12 provinces, and less than 1% in 14 provinces [87]. However, a diagnostic study conducted in 2014 in Northern Samar compared the prevalence determined using the gold standard KK method against qPCR results and found a discrepancy of nearly 70% (23% for KK vs. 90% for qPCR) [64], demonstrating the need for more sensitive diagnostic methods to determine true prevalence.

The Philippines experienced a period of success in controlling schistosomiasis after the launch of the Philippines Health Development Project (PHDP) in 1991, which was financially backed by a World Bank loan. The focus shifted to active case finding and mass drug administration with praziquantel; WASH interventions and snail control were included as additional measures. Drug coverage of the target population was reported to be 100% during the 1990s [76]. However, the prevalence of *S. japonicum* increased after the cessation of the program due to a lack of financial support; inadequate resources from the government also led to a diminished capacity to control schistosomiasis in the Philippines compared to P.R. China, where the government has strongly supported control efforts for over 50 years [17,183,184]. Due to insufficient funds to support the continuation of mass examination and treatment of at-risk populations, the Department of Health in the Philippines moved to targeting only high-prevalence endemic areas with mass drug administration [17,183].

In the Philippines, niclosamide has been banned for use in snail control under the Clean Water Act [17]. So, while mollusciciding has been performed in the past, special exemption would be needed for future use. A combination of methods employed in Japan was trialed on Bohol Island in the Philippines, focusing on grass cutting in swamps, followed by mollusciciding. Land reformation from swamps to rice fields, combined with mass drug administration, was effective in reducing prevalence to less than 1% [17]. In general, snail control measures successfully used in P.R. China have not been applicable to the Philippines due to differences in ecology and habitat of the local intermediate host snails [11]. Historically, environmental modification has been used in the Philippines to decrease snail habitats [127,185,186]. This rarely occurs now, and the mainstay of control in the Philippines is preventive chemotherapy with praziquantel [187]; yet, there is low compliance in taking the drug due to a variety of reasons, such as poor community engagement and fear of adverse events, which reduces the effectiveness of this intervention [82,188,189,190,191]. A cross-sectional survey undertaken in 2015 in Northern Samar, an area with high prevalence of schistosomiasis, reported treatment coverage of only 27% [28,190].

The success of P.R. China’s efforts since after the launch of the WBLP compared to the Philippines is largely attributable to the Chinese targeting both human and animals for chemotherapy as opposed to humans only in the Philippines, which ultimately proved to be far less effective [183]. The Philippines is currently undertaking measures to address the issue of *S. japonicum* infection in animals by strengthening veterinary health teams in priority areas through capacity building and operational research and moving toward implementation of WASH programs, as outlined during the 17th Meeting of the Regional Program Review Group on Neglected Tropical Diseases in the Western Pacific (175). These efforts are laudable and it can be anticipated that WASH will feature more prominently in the Philippines than in the past [187]. Lessons from early efforts (before the advent and wide use of praziquantel) to improve sanitation should be considered [82,96].

#### 4.1.4. Indonesia

In contrast to P.R. China and the Philippines, schistosomiasis in Indonesia is endemic in three comparatively small, isolated highland regions surrounding Lore Lindu National Park in central Sulawesi. These areas include marshes around Lake Lindu, particularly in the villages of Anca, Langko, Tomado, and Puro’o; Napu Valley [11] located 30–50 km southeast of Lake Lindu; and the more recently identified focus of Bada Valley [95]. Although the prevalence of *S. japonicum* has fluctuated over the last decade, prevalence tends to be higher in Napu Valley and overall appears to demonstrate an upward trend. Up to 2005, control efforts had decreased prevalence from 37% to 1% or less in Napu and Lindu valleys, but in the period from 2008 to 2011, prevalence varied between 0.3% and 4.8% in Napu and 0.8% to 3.2% in Lindu [95]. When Badu was first recognized as a new endemic area in 2008, prevalence was 0.5%, which later increased to 5.9% in 2010 [122].

Schistosomiasis control strategies set by the National Objectives for Health (NOH) directive for 2011–2016 varied based on the degree of endemicity, but overall took a multifaceted approach. Control objectives included mass drug administration coverage of 85% of the entire population in high endemic areas, active selective treatment in moderate endemic areas, and passive selective treatment in low endemic areas. Preventive chemotherapy has been supplemented with strategies that also focused on treating domestic animals, snail control, health education, improving water, and sanitation, and monitoring and evaluation and capacity building [76] in an effort to meet the 2020 elimination goal set by the Indonesian Ministry of Health [192]. Although Indonesia has made great strides in reducing the prevalence of schistosomiasis, control and prevention efforts have been inhibited by a lack of coordination and collaboration between the Ministry of Health and other ministries, as well as insufficient financial and human resources. The lack of funds dedicated to surveillance and control by the Ministry of Health may, in part, be due to the limited area and population affected by the disease or as a result of the decentralized and autonomous government system in Indonesia [193].

#### 4.1.5. Myanmar

Myanmar has been previously thought to be non-endemic for schistosomiasis, although there have been some historical unconfirmed reports of the presence of both *S. japonicum* and *S. mekongi* [16]. Recent studies have indicated that schistosomiasis has been emerging/re-emerging around Lake Inlay in central Myanmar [194]. Serological analysis of patient samples between 2012 and 2013 identified a prevalence of 23.8% (*n* = 315), whereas 302 cases were identified between 2016 and 2018 [16,195]. The WHO has been involved in supporting efforts to diagnose and treat infections in Myanmar, providing praziquantel, KK thick smear equipment, and urine tests [194]. Recently, molecular diagnostics determined a *S. mekongi* prevalence of 3.9% (*n* = 205) in the Bago Region of Myanmar [196,197].

A recent schistosomiasis outbreak occurred in Rakhine State with >400 confirmed cases and >800 suspected cases as of August 2018 [143]. Rakhine is the site of unrest due to political strife with Rohingya refugees and is also one of the poorest in terms of socioeconomic status in Myanmar, with a high number of households without access to clean water and proper sanitation [198]. This outbreak is occurring outside the area where schistosomiasis has previously been identified (Figure 1B). A technical team from the WHO and Myanmar Health and Sports Ministry have been to the area and suggested a special control team to carry out activities aimed at treating and preventing infection including health education in schools, diagnostics, treatment, and snail mapping [143]. The schistosome and snail species responsible for this current outbreak have not yet been identified.

It is not immediately clear from published reports if *S. japonicum* has been definitively diagnosed in Myanmar. Until the status of schistosomiasis is further clarified, ideally by molecular methods with which *S. mekongi* has already been confirmed, we rely on early reports that suggest both species are present [196,197].

### 4.2. S. mekongi

#### 4.2.1. Cambodia

The first case of schistosomiasis occurring in Cambodia was identified in 1968 in Eastern Cambodia and was seemingly confined to Vietnamese fishermen living in raft houses. Prevalence among children was higher (14–22%) than in adults (7–10%) [199]. At present, there are an estimated 80,000 people at risk of infection in Cambodia [142].

Control of schistosomiasis in Cambodia has been impacted by political unrest and upheaval through the 1970s and 1980s [200]. It was not until 1993 that control programs targeting schistosomiasis commenced. At the onset of the control program, drug compliance was low due to the treatment not being free. Subsequently, mass drug administration was provided free of charge [200]. This approach has been the mainstay of control in Cambodia for the last 20 years and, in 2016, the schistosome prevalence determined by the KK thick smear technique was 0%. In addition to preventive chemotherapy, CL-WASH programs were implemented in endemic villages in 2016. Facilitators were initially trained in CL-WASH, who then helped lead community-based training when education on schistosomiasis transmission was provided and linked to sanitation and hygiene habitats. In each community, CL-WASH teams, composed of volunteers from each community as well as a facilitator, conducted surveys on village households to determine the provision of sanitation and the level of malnutrition [201]. Initial surveys found that >60% of households did not have toilets and many still practiced open defecation. Survey results were mapped and presented to the community members who discussed how their behaviors led to schistosome infection (and other parasitic worm and intestinal protozoa infections) and how infection could be prevented with the development and implementation of CL-WASH plans. These plans included the building and use of latrines in villages [201]. Elimination was planned for 2017, although this was reliant on more sensitive diagnostic tools being employed, an increase in sentinel site surveillance, and increased use of CL-WASH in endemic areas [202].

#### 4.2.2. Thailand

The first case of *S. mekongi*, described as a *S. japonicum*-like infection, was reported in 1950 in Thailand [199]. This ‘patient zero’ was a Thai native and thus it is likely this was an autochthonous case. Further investigations identified an endemic region and susceptible intermediate host snail in southern Thailand. In 1964, further cases were identified in northern Thailand. Animals including water buffalo, cattle, dogs, cats, pigs, and rats were also surveyed in the endemic areas but none were positive at that time [199]. Thailand is currently in transmission interruption, waiting on investigation and ratification by the WHO [203].

#### 4.2.3. Lao PDR

Lao PDR followed Thailand in the identification of *S. mekongi* in 1957 in a patient who had been living in Paris for nine years, but had spent the first nine years of life in Lao PDR [34,199]. This observation indicated that the adult worms can live for many years. The patient was admitted with what was thought to be cirrhosis of the liver; a liver biopsy revealed the presence of a *S. japonicum*-like egg. It was not until 1966 that a second case was identified in Lao PDR and, in 1969, an epidemiological survey determined a prevalence of 14.4% (*n* = 72) on Khong Island. As with *S. japonicum*, hepatosplenomegaly was associated with infection [34,199,204]. On Khong Island, the parasite was first found in animals, specifically in a dog [34].

A study examining the prevalence of a range of parasites in islands of the Mekong in Lao PDR performed in 2011 determined a prevalence of 22.2% (*n* = 994) in humans and 14.7% (*n* = 68) in dogs for *S. mekongi* [123]. The infected snail prevalence was very low with 0.01% (*n* = 29,583) of collected snails being positive [123]. More recent cases have been identified in returning travelers who visited these historically endemic areas, including a Belgian visitor to Khong Island in 2013 and a French woman who was exposed to freshwater habitats in southern Lao PDR [205,206]. These cases indicate that transmission is ongoing.

Mass drug administration in Lao PDR was first carried out yearly from 1989 to 1998. In the first year of the program, only selective chemotherapy was practiced but this was expanded in subsequent years [200]. Before the onset of this preventive chemotherapy-based program, there were an estimated 11,000 cases with a further 60,000 deemed to be at risk of infection. By 1999, the prevalence was significantly reduced, but after cessation of yearly mass drug administration, the prevalence rebounded to pre-intervention levels. Hence, mass drug administration was recommenced in 2007 with financial support from WHO [202]. CL-WASH was implemented in 10 villages in 2016, to be expanded to all endemic villages by 2020 [202].

#### 4.2.4. Myanmar

See Section 4.1.5. for Myanmar under *S. japonicum*.

### 4.3. S. malayensis

#### Malaysia

Schistosomiasis resembling *S. japonicum* was first identified in Malaysia in 1973 during an autopsy [199]. Previous schistosome infections identified in Malaysia were *S. japonicum* in foreign nationals from P.R. China and Singapore [207]. A review of autopsy materials between 1967 and 1975 uncovered a further nine cases of *S. japonicum*-malaysia (later classified as *S. malayensis* in 1988) [199,207]. No animal infections, snail hosts, or new human cases in the areas that the infected deceased came from could be identified [199]. The strain that caused schistosomiasis in these cases was thought to be different from the Mekong schistosome [199], and likely represents the first case of *S. malayensis.*

Until 1978, all cases of *S. malayensis* were identified in patients who were deceased with schistosomiasis identified in autopsies, although not as the reported cause of death [207,208]. In general, cases of *S. malayensis* have been aboriginal Malaysians (Orang Asli) living in rural areas and patients were either previously or concurrently co-infected with other infectious diseases. The first case identified in a living patient occurred in Selangor State and the patient presented with hepatosplenomegaly [207,208].

In the meantime, *S. malayensis* has only been recorded sporadically in humans, with few recent accounts of infected individuals. The most recent identification of infection in humans was reported in a pathology report of an individual in 2011 [209]. In this case, histology slides of the liver identified liver granulomas, although schistosomiasis had not been diagnosed prior to the death of the patient [209].

## 5. Concluding Remarks

Multi-component, intersectoral, and integrated control approaches provide a promising path forward for the elimination of schistosomiasis in Asia. Behavioral changes that prevent infection, such as avoiding the practice of open defecation and contact of open freshwater bodies in endemic areas, are necessary. However, without accompanying infrastructure, such as toilets provided in WASH programs, these behaviors will continue. Combined with chemotherapy of both humans and reservoir hosts (e.g., water buffalo), snail control, animal vaccination, health education, and WASH targeting multiple points in the schistosome life cycle will significantly impact the prevalence and re-infection of schistosomiasis. The success achieved in P.R. China in schistosomiasis control and elimination is due to the commitment and support of the Chinese government, including the removal of water buffalo—a major reservoir host in P.R. China—from endemic areas, thus effectively removing them from the transmission cycle. Without such governmental support, sustaining control programs is difficult, particularly those that include more than preventive chemotherapy and require considerable resources to implement, such as required for CL-WASH. There is a niche role for health education that is missing from many schistosomiasis control programs, as knowledge remains limited about the parasite and which behaviors lead to infection in endemic populations.

Limited data are available on prevalence for *S. mekongi*, particularly for *S. malayensis*, the number of human cases, and the role played by animal reservoirs in transmission. Thus, assessing the true importance of schistosomiasis in the countries where these two schistosome species are endemic is difficult.

The true *S. japonicum* prevalence is conceivably underestimated, both in P.R. China and the Philippines, due to the lack of sensitive diagnostics used in control programs. While P.R. China closes in on elimination targets, the use of sensitive diagnostics will be important to determine whether elimination has indeed occurred and to prevent re-bounding infections after treatment and the cessation of the control program. A recent study in P.R. China found a human prevalence <1% by the MHT but 11% by qPCR [63]. Although the majority of the cases detected by qPCR were light-intensity infections, they do present a significant number of individuals who were not identified by widely used diagnostic methods, and who could contribute to resurgence after interventions cease. Limited case finding and prevalence studies have been performed in either country by their national control programs. Another limitation is the lack of snail prevalence surveys performed in many of the schistosome-endemic countries.

Future challenges for schistosomiasis control and elimination include climate change and the potential spread of the disease to new areas [141,210,211,212]. Europe has, for example, seen a return of autochthonous schistosomiasis cases, although this may initially be due to human migration from endemic areas [213]. Increases in temperature due to climate change will shift the tropical zone, the band in which schistosomiasis currently occurs. In P.R. China, this may lead to a shift in endemic areas further North in the country as the climate changes, and with the implementation of the SNWD project.

## Figures and Tables

**Table 1:** A comparison of features of the three Asian schistosome species that can infect humans [4,25,26].

	Geographic Distribution	Animal Definitive Hosts	Intermediate Hosts	Eggs
***S. japonicum***	Indonesia, the Philippines, P. R. China	46 known mammalian hosts including water buffalo and cattle, dogs, pigs, and rodents	*Oncomelania* spp. 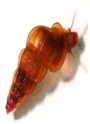	70–100 × 55–64 µm 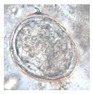
***S. mekongi***	Cambodia, Lao PDR, Thailand	Dogs and pigs	*Neotricula* spp. 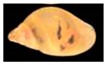	50–80 × 40–65 µm 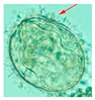
***S. malayensis***	Malaysia	Rodents	*Robertsiella* spp. 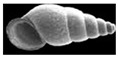	53–90 × 33–62 µm 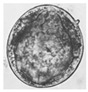
